# Beyond Usage Time: Rethinking Therapeutic Dose and Effect in Digital Therapeutics

**DOI:** 10.2196/98660

**Published:** 2026-08-14

**Authors:** Björn Meyer, Linda T Betz, Mario Weiss, Wolfgang Lutz

**Affiliations:** 1 Research and Development, GAIA (Germany), Hans-Henny-Jahnn-Weg 53, Hamburg, Hamburg, 22085, Germany, 49 17610392337; 2 GAIA (Germany), Hans-Henny-Jahnn-Weg 53, Hamburg, Hamburg, 22085, Germany; 3 Division of Clinical Psychology and Psychotherapy, University Trier, Trier, Rheinland-Pfalz, Germany

**Keywords:** digital therapeutics, therapy duration, dose-response relationship, mental health, engagement, personalization

## Abstract

Digital therapeutics (DTx) have become increasingly prominent in mental health care, offering scalable, evidence-based interventions. A common assumption underlying their design and evaluation is that greater usage time leads to superior therapeutic outcomes, reflecting an implicit linear dose-response model. However, accumulating evidence challenges this simplified perspective and suggests that the relationship between usage time and clinical benefit is substantially more complex. In this viewpoint article, we critically examine the assumption that higher usage time is a necessary prerequisite for therapeutic success in digital mental health interventions. Drawing on psychotherapy dose-effect research, findings from digital intervention trials, and our own empirical work, we highlight several factors that complicate linear exposure models. These include rapid early response and plateau effects, substantial heterogeneity in user trajectories, motivational and contextual determinants of use, episodic patterns of engagement, and the distinction between on-platform activity and off-platform skill application. The linear dose metaphor, implicitly inherited from pharmacotherapy models, appears conceptually mismatched with learning-based digital interventions. Importantly, equating greater use with greater effectiveness risks conflating exposure with therapeutic mechanism, and may inadvertently promote evaluation frameworks that prioritize duration over meaningful change. Together, these insights suggest that usage time is an imperfect proxy for therapeutic engagement, and that high or continuous use should not be treated as a universal indicator of intervention quality. We argue for a shift toward mechanism-informed and individualized evaluation frameworks that prioritize the quality, timing, and functional impact of intervention use over cumulative exposure.

## Is Usage Time Really the Most Meaningful Indicator of Quality in Digital Therapeutics?

Digital therapeutics (DTx) are evidence-based interventions delivered via digital platforms to prevent, manage, or treat mental health conditions. In mental health care, DTx are typically based on cognitive behavioral therapy and offer scalable, accessible solutions for conditions such as depression, anxiety, and stress-related disorders [1]. The widespread adoption of smartphones and increased internet accessibility have facilitated the proliferation of these interventions, expanding access to underserved populations.

A prevailing assumption in both research and clinical practice is that higher usage time of a DTx (ie, greater exposure) is directly proportional to better therapeutic outcomes, consistent with a presumed linear dose-response model [2-7]. This perspective suggests that the more time a user spends using the intervention, the greater the expected improvement in symptoms and related outcomes. Consequently, considerable efforts have focused on increasing usage time through reminders, gamification strategies, and incentive structures [5,8].

In this viewpoint article, we argue that such a quantity-focused view of therapeutic exposure oversimplifies the mechanisms of change in digital interventions. Building on interdisciplinary research and our own empirical work, we critically examine the assumption of a linear dose-response relationship in psychotherapy provided by DTx. Our discussion integrates findings from psychotherapy dose-effect research, digital mental health trials, and trajectory-based analyses to highlight key conceptual considerations rather than to offer an exhaustive review.

Our argument is founded on three challenges to the “more is better” paradigm: (1) the ubiquity of nonlinear change patterns in digital and nondigital psychological therapy, (2) the importance of contextual predictors of treatment engagement, and (3) the primacy of facilitating “offline skill acquisition” rather than maximizing “time spent online.” Our aim is to articulate why usage time is an inadequate proxy for therapeutic engagement and to propose a multidimensional, mechanism-informed framework—distinguishing exposure from therapeutic effect—to guide how DTx are designed and evaluated.

## Redefining the Dose: Distinguishing Exposure From Therapeutic Effect

The intuition that “more is better” is not, in itself, a flawed application of dose-response logic. Dose-response thinking remains foundational across medicine, and abandoning it would be neither necessary nor desirable. The problem is narrower and more specific: in DTx, dose has been operationalized almost entirely as usage time, and usage time is a poor stand-in for the quantity that actually drives change. The issue is therefore not dose-response reasoning but a dose that has been defined too narrowly.

This distinction is clarified by the way pharmacology already separates the dose that is administered from the dose that ultimately acts. A prescribed dose must be absorbed (bioavailability), reach a sufficient concentration at its site of action, and only then produce a response—and each of these transitions is nonlinear, shaped by thresholds, saturation, and substantial interindividual variability. Time spent holding a tablet is not the active dose. By analogy, time spent using an application is not the therapeutic dose of a digital intervention; it is merely the most readily logged event in a longer causal chain.

**Figure 1.**
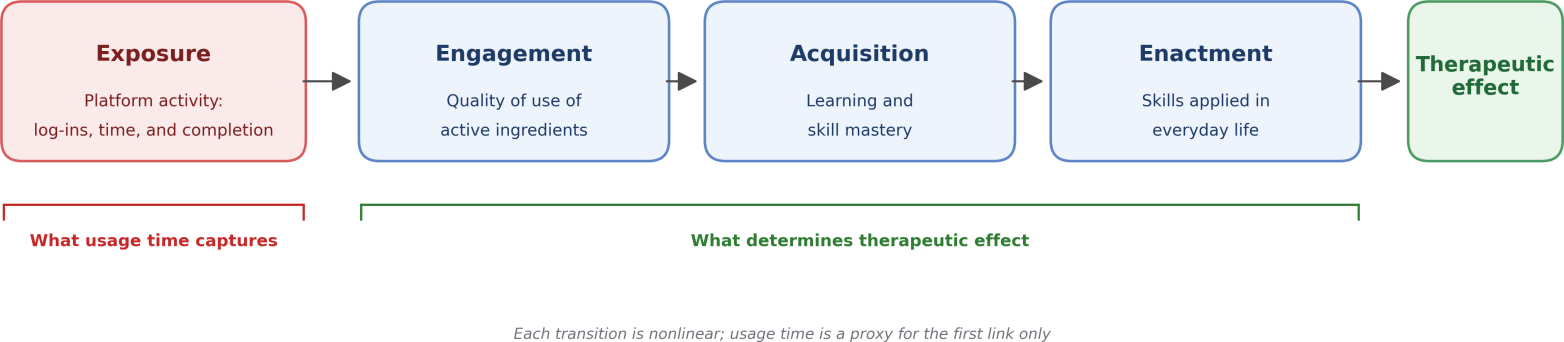
The therapeutic exposure cascade in digital therapeutics. Usage time captures only the first link (exposure); the quality of engagement, skill acquisition, and real-world enactment determine the therapeutic effect, and each transition is nonlinear.

We therefore distinguish 4 sequential stages through which a digital intervention produces benefit (Figure 1). Exposure is observable platform activity—log-ins, session duration, and completion—which is what current engagement metrics overwhelmingly capture. Engagement is the quality of interaction with the active therapeutic ingredients rather than its volume: whether a user works through a cognitive exercise meaningfully, not merely whether they opened it. Acquisition is the learning and skill mastery that constitute the mechanism of change. Enactment is the application of those skills in everyday life beyond the platform—what has been termed macroengagement [9]. Therapeutic effect follows from this final stage. Usage time is a proxy for the first link only, and an increasingly leaky one: each downstream transition is nonlinear, and the benefit that matters most often accrues at the enactment stage, where platform analytics are by definition blind.

Reframed this way, the phenomena we examine below are not a loose collection of exceptions to a dose-response rule but predictable features of a multidimensional one. Early response reflects rapid traverse of the cascade; plateau and “good enough” effects reflect saturation at the effect link, beyond which further exposure adds little; episodic, as-needed use reflects adaptive redosing rather than disengagement; and off-platform benefit reflects the cascade completing where it is hardest to observe. Dose still matters—but its relevant dimensions are quality, timing, target, and enactment, not cumulative duration.

This reframing also carries a cautionary edge. When usage time is treated not as an imperfect proxy but as an optimization target—engineered upward through reminders, gamification, and incentives, or rewarded directly in evaluation and reimbursement frameworks—it becomes vulnerable to a familiar failure: a measure optimized for its own sake ceases to track the goal it was meant to represent. High use may reflect unmet need, distress, or even dependence rather than effective treatment, and rewarding it risks incentivizing designs that maximize time-on-platform at the expense of efficient therapeutic benefit. Treating usage time as one weak signal within a multidimensional cascade, rather than the headline indicator of quality, is therefore not only a conceptual correction but a safeguard against perverse incentives in how DTx are designed, evaluated, and valued.

Throughout, we use exposure for observable platform activity, engagement for the quality of interaction with active therapeutic ingredients, adherence for use relative to a prescribed or recommended amount, and usage time specifically for the cumulative duration of platform activity. These terms have often been used interchangeably in the literature—an imprecision that has itself contributed to the conflation we critique.

## Symptom Change Rarely Follows Linear Patterns

A first and central challenge to the linear dose-response assumption is that symptom change in psychotherapy—including psychotherapy provided by DTx—is frequently nonlinear. Many patients experience substantial gains early in psychotherapy, followed by stabilization or plateau phases. Evidence confirms that therapeutic change typically follows a negatively accelerating pattern, such that symptomatic improvement is most pronounced during initial sessions and then diminishes as patients approach a plateau. Indeed, fewer than 20% of patients tend to exhibit a linear treatment response, and there is no universal “optimal therapeutic dose” that suits all patients, given that patients differ in impairment, comorbidity, treatment responsiveness, and other relevant characteristics [10].

The nonlinear nature of change is illustrated by therapeutic phenomena such as early gains and subsequent plateau effects. Gains in psychotherapy often stabilize once a clinically meaningful threshold has been reached [11]. Beyond this point, additional use tends to yield minimal added benefit [11-13]. Pushing for continued use beyond this point may not improve outcomes and can even contribute to fatigue or disengagement, particularly in digital interventions that lack personalization [14].

Such temporal dynamics make it clinically reasonable and efficient for patients and therapists to discontinue or reduce intervention exposure once sufficient improvement has been achieved. Psychotherapy research has long documented these early response patterns, often referred to as rapid responders [11,15,16]. Meta-analytic evidence suggests that similar early gains in digital interventions are associated with sustained long-term benefits [17]. For these patients, prolonging treatment long beyond adequate early response would be implausible and inefficient.

Evidence from our randomized controlled trial evaluating *deprexis* supports this pattern: many program users exhibited clinically meaningful reductions in depressive symptoms within the initial weeks of treatment, irrespective of full program completion [18-20]. These early improvements were linked to sustained benefits, even over a 3-year period [21].

These findings align with broader developments in precision medicine, which emphasize response heterogeneity and the tailoring of interventions to individual trajectories. Psychotherapy research has shown that response patterns differ systematically and can be predicted for individual patients. This allows for the identification of “on track” (ON) versus “not on track” (NOT) cases and corresponding treatment modification, such as altering the type, intensity, or frequency of treatment components [10].

The general promise of such responsive treatment allocation is that therapy can be delivered more effectively and efficiently when progress with respect to a patient’s actual versus expected progress is monitored and adjusted dynamically [10]. Rather than providing a standard dose for all, then, digital as well as nondigital psychological treatments ought to be deployed responsively as patients align with or deviate from their predicted progress paths.

Trajectory analyses of DTx are consistent with this idea as they have shown that distinct response subgroups can be identified, including patients who demonstrate rapid and pronounced early improvement and derive limited additional benefit from extended usage time. For these individuals, continued exposure may produce diminishing returns rather than incremental therapeutic gains [22]. Taken together, these findings indicate that simply maximizing usage time without attending to individual progress trajectory is misguided and may even be counterproductive. The goal of digital or nondigital therapy is alleviating suffering and restoring mental health efficiently, after all, rather than pushing patients to engage with therapy as an end in itself. Such use maximization efforts could even lead to addiction-like or compulsive patterns, as the potential for problematic or compulsive patterns of software use has been documented in broader digital health contexts and warrants careful consideration in intervention design [23-25].

## Early Response and the “Good Enough” Effect

The contrast with pharmacotherapy is instructive but should not be overdrawn. Contemporary pharmacology does not assume a simple linear dose-response relationship; pharmacometric models routinely incorporate thresholds, saturation (ceiling) effects, and substantial interindividual variability in how an administered dose translates into exposure and ultimately response. What distinguishes learning-based digital interventions is therefore not the presence of nonlinearity per se, but the nature of the active ingredient: mechanisms of change operate through learning, skill acquisition, and cognitive restructuring—processes that can reach functional sufficiency well before maximal exposure is achieved and whose effective “dose” is consequently poorly indexed by time. The distinction between an administered dose and the dose that ultimately acts applies here with even greater force.

The “good enough” effect, well established in psychotherapy research, offers a coherent explanation for such patterns. Individuals often discontinue treatment once sufficient improvement has been achieved, regardless of the a priori intended duration [11,26]. Rapid early response and subsequent plateau effects create conditions in which reducing or discontinuing intervention exposure can represent a rational and adaptive decision. In this context, lower usage time may reflect successful skill acquisition and recovery rather than insufficient engagement. Discontinuation does not necessarily indicate treatment failure but may instead signal that therapeutic goals have been sufficiently met [27,28].

Evidence from randomized controlled trials evaluating *deprexis* supports this interpretation: participants demonstrated clinically meaningful symptom reductions even when they discontinued usage before completing the full program [19,29]. Other studies likewise report that greater usage time does not consistently yield superior therapeutic outcomes [3,30-39].

Taken together, these findings suggest that therapeutic benefit does not scale proportionally with usage time. Many individuals reduce or discontinue use precisely because meaningful improvement has already occurred. In this sense, declining use may reflect progress rather than disengagement. Yardley et al [9] describe a shift from maximal to effective engagement, emphasizing the amount of use required to achieve meaningful benefit rather than adherence for its own sake. This distinction is particularly important in digital mental health, where intervention use must be balanced with everyday demands and where excessive or unnecessary exposure may carry unintended consequences, including the risk of maladaptive or compulsive use patterns in vulnerable populations.

## Treatment Use Has Individual and Contextual Determinants

A second relevant challenge to the “more is better” paradigm concerns the contextual nature of treatment use. That is, patients differ widely in their life contexts, including not only their symptomatic characteristics but also personality and social contexts, including attitudes, needs, and preferences, life stressors, social support, and other dimensions. Effective treatments—digital or not—should not ignore these multifaceted contextual factors but should instead seek to adapt and tailor interventions to them. Therefore, merely maximizing treatment dosage regardless of context is misguided.

Consistent with this idea, evidence indicates that personalized or adaptive interventions—those that tailor content to individual needs—are associated with improved outcomes and more efficient use [40-44]. By contrast, standardized programs that require all users to progress through identical content, regardless of relevance, risk misalignment with individual needs. In such cases, increasing usage time or insisting on full completion does not necessarily enhance therapeutic benefit; it may instead dilute relevance and reduce efficiency [14,38,40,42].

These person-level differences are closely intertwined with motivational processes that shape how much usage time is appropriate or sustainable. Intrinsic motivation plays a central role in digital intervention use [5]. When patients perceive content as meaningful, personally relevant, and aligned with their goals, they tend to process the information more thoroughly and engage more actively with the intervention, often facilitating effectiveness [41,45]. Enhancements in user experience, such as gamification and interactive features, can support perceived relevance and intrinsic motivation without necessarily prolonging usage time [1,46,47]. Supporting user autonomy and aligning intervention content with personal goals further promotes meaningful engagement and strengthens therapeutic impact [39]. Supporting autonomy and aligning intervention content with personal goals may therefore strengthen therapeutic impact more effectively than simply increasing usage time.

Beyond motivation, patients’ capacity for intervention use is shaped by fluctuating life circumstances, including everyday demands and environmental stressors [48]. These contextual factors influence not only the opportunity for use but also therapeutic responsiveness. Empirical findings support this view. Data from the EVIDENT trial of *deprexis* indicate that life events and stressors significantly influenced both use patterns and outcomes [18]. For some individuals, competing responsibilities limit capacity for sustained use; for others, acute stress may temporarily increase reliance on the intervention. Such variability further challenges the assumption that uniform use targets are appropriate across individuals and contexts [4,39,49].

Moreover, time spent using a digital intervention may compete with other adaptive or health-promoting activities, creating opportunity costs that limit the feasibility or desirability of increased use. In some contexts, encouraging higher use without considering individual circumstances may be counterproductive. Expectations that exceed individuals’ situational capacities can elicit reactance or feelings of inadequacy, thereby reducing rather than enhancing meaningful intervention use [14].

Beyond individual and contextual influences, temporal patterns of use vary substantially across patients [50]. A growing body of research indicates that intervention use is often episodic rather than continuous, with many individuals returning to a digital therapeutic “as needed” in response to acute stressors, specific challenges, or periods of heightened symptom burden [9,51].

Conceptually, many DTx are designed to function in this flexible manner—providing support when required rather than requiring sustained, intensive use over extended periods [52,53]. For such users, episodic and context-driven use may represent the most appropriate and effective “dose” of the intervention. This flexible, on-demand pattern sits in tension with regulatory and reimbursement frameworks that tie value to rigid engagement thresholds—for example, classifying users as dropouts after only a few inactive weeks—which risk penalizing the very usage patterns that DTx are designed to enable.

These temporal dynamics are reflected in empirically observed usage profiles. Large-scale analyses show that DTx routinely attract distinct user subgroups, including low engagers, late engagers, and users with high initial use followed by rapid disengagement. Notably, this latter group improved more despite lower overall use than groups with higher use, indicating that the timing of use may be more consequential than its duration, with early engagement potentially benefiting from heightened motivation or readiness for change [50]. Conversely, very high levels of use may in some cases reflect elevated distress or unmet needs rather than effective therapeutic engagement. Greater usage time is therefore not inherently indicative of clinical progress.

## The Quality and Location of Engagement

A third fundamental challenge to the “more is better” paradigm is that online activity (“usage time”) may reflect interaction with the digital interface but does not necessarily capture engagement with the underlying behavior change processes that drive therapeutic improvement [8]. DTx are not designed merely to increase time spent within an application; rather, they aim to facilitate meaningful therapeutic learning and “offline skill acquisition.” Clearly, the goal of treatment is to empower patients with skills to improve their quality of life and well-being beyond the digital realm rather than spending more time online.

This points to a more fundamental distinction: DTx are delivery systems for therapeutic mechanisms, not therapeutic mechanisms in themselves. The active ingredient is the cognitive or behavioral change the intervention teaches, not the software through which it is taught. Just as the effect of a medication lies in its pharmacological action rather than in the act of swallowing a tablet, the benefit of a DTx lies in the skills users acquire and enact, not in the time spent within the application. Measuring platform activity therefore indexes the vehicle rather than the mechanism—a mismatch that becomes most visible precisely when interventions succeed, because users who have internalized the relevant skills require less, not more, contact with the software.

Many interventions explicitly focus on teaching skills that users can apply independently in their everyday lives. Once such skills have been acquired, continued online use may become less central to therapeutic progress. In this context, reductions in usage time can reflect successful skill acquisition rather than disengagement [9]. Empirical findings support this interpretation: even brief digital interventions can yield sustained improvements when they effectively transmit coping strategies that users subsequently apply independently in real-world settings [54,55].

From this perspective, “macroengagement”—the enactment of intervention-derived skills in everyday life—may be a more meaningful indicator of therapeutic success than the quantity of tracked platform interactions [9]. Many DTx explicitly support this off-platform orientation by providing worksheets, audio exercises, summaries, or prompts designed to encourage autonomous skill use beyond the application environment. Such design features deliberately decouple therapeutic benefit from platform activity, implying that variable or declining on-platform use does not inherently signal diminishing effectiveness.

Human support can operate within this broader framework by enhancing the quality and timing of therapeutic engagement rather than simply increasing its duration. Brief guidance—such as structured check-ins or motivation-focused feedback—has improved outcomes in several digital interventions [29,56], although effects are not uniform across studies [57]. Importantly, such support appears to strengthen meaningful or strategically timed use rather than merely prolonging exposure.

Taken together, effective engagement often unfolds in ways that platform analytics cannot capture. Substantial progress may occur when users apply acquired skills independently or reengage at personally meaningful moments. Accordingly, lower or fluctuating usage time does not necessarily indicate reduced effectiveness but may instead reflect adaptive integration of therapeutic learning.

## Implications for the Future of DTx

This viewpoint has advanced one linked argument across 3 challenges: therapeutic change in DTx is nonlinear, is contextually determined, and is frequently realized off-platform, so that usage time captures only the first and weakest link in the chain from exposure to therapeutic effect. We challenge the persistent assumption that more intensive use necessarily produces superior outcomes or should be a primary goal in DTx for mental health. Across diverse lines of evidence, a consistent pattern emerges: therapeutic change in digital interventions is nonlinear, heterogeneous, and context dependent. Accordingly, high usage time should not be regarded as a prerequisite for success nor as a reliable marker of intervention quality or an intrinsically valuable process goal.

The empirical and conceptual considerations outlined in this article indicate that reductions in use often reflect adaptive processes rather than failure. Early responders, individuals who reach a personally sufficient level of improvement, and users who internalize skills and shift toward off-platform application all demonstrate that therapeutic benefit does not necessarily require prolonged or complete program exposure. Plateau effects, contextual constraints, and episodic usage patterns further challenge the implicit benchmark that full intervention completion represents optimal care.

A central implication is that quality of engagement matters more than quantity. Personalized and adaptive systems are therefore better positioned to deliver clinically meaningful benefits than rigid structures built on uniform dosage assumptions. Undifferentiated efforts to increase usage time may misallocate resources, increase user burden, and divert attention from the mechanisms through which change actually occurs.

For research and evaluation, these findings call for a shift toward multidimensional, mechanism-sensitive metrics that extend beyond on-platform activity. Measures focused solely on log-ins, completion rates, or aggregate usage time risk conflating exposure with therapeutic process and obscuring meaningful heterogeneity in patient trajectories. Future research should identify which subgroups benefit from sustained use, for whom early or intermittent contact is sufficient, and how digital interventions can dynamically adapt to these differing needs. Digital phenotyping and machine learning approaches hold promise for identifying such subgroups and informing treatment allocation and modification procedures in clinical practice [10]. In psychotherapy research, adaptive feedback systems are being developed, which alert clinicians and patients when progress deviates from expected paths. For example, problems with the alliance, motivational issues, emerging life events, or other critical factors that require treatment adaptations are flagged, opening opportunities for dynamic intervention adjustments to optimize outcomes [10]. Digital treatments are ideally suited to follow this logic, given that progress indicators are logged continuously, and continuously updated algorithms can be used to tailor interventions to dynamically changing patient needs. Psychological treatments—digital or not—ought to be responsive to such evolving patient needs and be delivered in a form, intensity, and frequency that matches individual patient requirements rather than at a fixed or universal maximum dosage.

These commitments can be made concrete. If usage time is a weak proxy for only one link in the cascade, the constructive task is to specify what a more complete measurement model would capture. We do not propose a single replacement metric; the heterogeneity described above makes that neither realistic nor desirable. Instead, evaluation should move from a 1D volume measure toward a small set of complementary indicators aligned with the stages through which interventions actually work.

At the level of engagement quality, instrumentation can distinguish active from passive use—for example, completion of core therapeutic tasks such as a thought record or an exposure exercise, rather than module opens or screen time alone, together with the depth and consistency of interaction with specific active ingredients [8,9]. At the level of skill acquisition, brief in-application assessments of comprehension, competence, or mastery can index whether the mechanism of change is being engaged, not merely whether content was delivered. At the level of enactment—arguably the most consequential and least measured stage—ecological momentary assessment, skill use logs, and passive behavioral sensing (digital phenotyping) can capture whether learning is applied in daily life, where platform analytics are blind [10,58-60].

Outcomes themselves should be framed in trajectory terms rather than as end point snapshots. Methods for modeling a patient’s expected treatment response and classifying cases as on track or not on track are well developed in psychotherapy research and transfer naturally to DTx, where progress indicators are logged continuously [10,22]. Such models permit early identification of rapid responders, plateaued users, and patients deviating from expected paths—each of which implies a different, and sometimes lower, optimal level of further exposure. Functional outcomes and quality of life, not symptom scores alone, should anchor these trajectories.

Together, these indicators enable responsive, adaptive dosing rather than uniform exposure targets. Continuously logged progress can drive just-in-time adaptive interventions and measurement-based feedback that adjust the type, intensity, and timing of content to a patient’s evolving needs [10,52,53]. Digital phenotyping and machine learning are well suited to this task, but their value depends on first defining the targets they are meant to predict.

This identifies the field’s central open problem: progress toward “good” outcomes cannot be measured without first defining them. We therefore see the most valuable next step as the development of empirically grounded outcome and trajectory typologies through triangulation of data-driven (eg, machine learning–derived response subgroups), theory-driven (mechanism-based), and clinically grounded (pragmatic and patient-centered) approaches. Convergence across these perspectives would yield reference trajectories against which meaningful engagement—rather than mere exposure—could be defined and benchmarked.

In conclusion, the field must move beyond the reflexive assumption that “more is better.” Effective DTx are not defined by maximal exposure but by the timely delivery and successful integration of therapeutic skills. Recognizing when additional use is beneficial—and when it is unnecessary—will support the development of interventions that are both clinically effective and aligned with the realities of human behavior.

## Abbreviations

DTx: digital therapeutics
